# Event-related potentials during encoding coincide with subsequent forced-choice mnemonic discrimination

**DOI:** 10.1038/s41598-024-66640-7

**Published:** 2024-07-09

**Authors:** Leslie Rollins, Alexis Khuu, Kaylee Bennett

**Affiliations:** Department of Psychology, Christopher Newport University, 1 Avenue of the Arts, Newport News, VA 23606 USA

**Keywords:** Mnemonic discrimination, ERP, Subsequent memory effect, Encoding, Encoding variability, Long-term memory, Cognitive neuroscience

## Abstract

Computational models and eye-tracking research suggest that encoding variability accounts for the reduced recognition of targets (A) when paired with non-corresponding lures (B′) relative to corresponding lures (A′). The current study examined whether neural activity during learning coincided with subsequent performance on the forced-choice Mnemonic Similarity Task (MST). Event-related potential responses were collected during encoding while young adults completed A–B′ and A–A′ trials of the forced-choice MST. Consistent with previous research, performance was lower on A–B′ trials than A–A′ trials. The subsequent memory effect was not significant for the A–A′ test format. However, for A–B′ trials, we observed a significant Accuracy × Stimulus interaction 1000–1200 ms poststimulus onset across frontal and fronto-central electrodes. As hypothesized, subsequently correct A–B′ trials were associated with a larger amplitude response at encoding to the target (A) than the original version of the non-corresponding lure (B). However, subsequently incorrect trials were associated with a larger amplitude response to the non-corresponding lure (B) than the target stimulus (A). These findings provide additional support for the effect of encoding variability on mnemonic discrimination.

Pattern separation and pattern completion are fundamental processes that underlie the ability to discriminate between similar memory representations^1^. Pattern separation is a computational process that reduces interference between overlapping representations whereas pattern completion supports the retrieval of stored memory representations when provided with partial or degraded input (e.g.,^2,3^). As such, ineffective pattern separation during encoding can result in subsequent mnemonic discrimination errors^4,5^. Mnemonic discrimination is commonly assessed using the Mnemonic Similarity Task (MST, for review see^6^). During the forced-choice version of the MST, a target is either presented with a novel object (A–X), a corresponding lure (A–A′), or a non-corresponding lure (A–B′). Performance on the forced-choice MST is highest for the A–X test format, intermediate for the A–A′ test format, and lowest for the A–B′ test format^7–9^. Application of the global matching model MINERVA 2 (Hintzman 1984, 1988) by Huffman and Stark^7^ suggested that encoding variability could partially account for reduced performance on the A–B′ test format relative to the A–A′ test format. Specifically, if the B stimulus was more effectively encoded than the A stimulus, the non-corresponding lure (B′) may elicit a stronger global match in memory than the target (A). This proposal has been empirically supported by eye-tracking studies; errors on A–B′ trials are associated with more fixations to the B stimulus than the A stimulus at encoding^9,10^.

The goal of the current study was to examine whether neural activity during learning coincided with subsequent performance on the forced-choice MST. Event-related potentials (ERPs) have provided insight into processes that support effective mnemonic discrimination at retrieval^11–15^ and may be able to further understanding of how encoding variability influences mnemonic discrimination. Previous research has revealed that electrophysiological activity at encoding differentiates subsequently recognized and forgotten items (for reviews see^16,17^). Mecklinger and Kamp^17^ recently identified that subsequent memory effects (SMEs) can be divided into three separate subcomponents. Early (300–500 ms) frontal and parietal SMEs reflect the encoding of verbal information and feature binding, respectively, whereas the prolonged late-onset frontal SME reflects associative encoding.

For the current study, young adults completed a modified forced-choice MST that included the A–A′ and A–B′ test formats while electroencephalography was recorded during encoding. We hypothesized that individuals would show greater accuracy on the A–A′ test format relative to the A–B′ test format. Further, we anticipated that incorrect A–B′ trials would be associated with a higher mean amplitude to the B stimulus than the A stimulus during encoding, whereas correct A–B′ trials would be associated with a higher mean amplitude to the A stimulus than the B stimulus during encoding. Although encoding variability typically exerts a weaker effect on A–A′ performance^7,9^, we also assessed whether a subsequent memory effect would emerge in this test condition as well, which would be reflected by a higher amplitude response to subsequently correct than subsequently incorrect trials.

## Results

### Behavioral results

**Figure 1:**
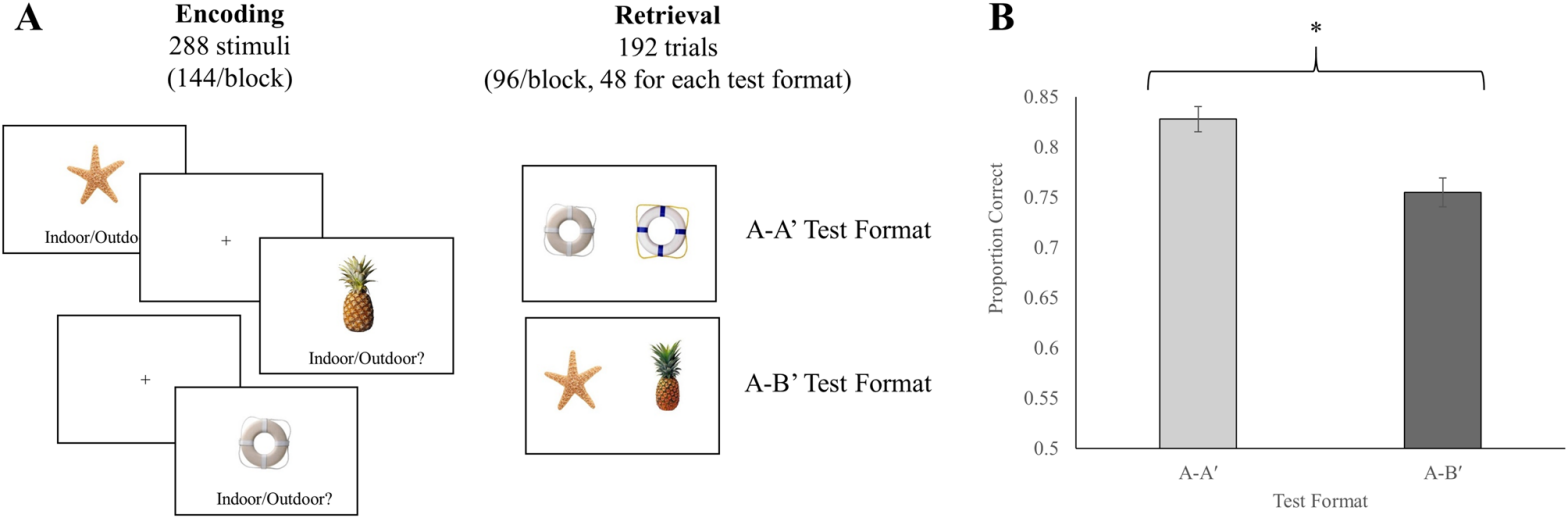
(**A**) Schematic of the experimental design depicting sample stimuli and test formats (**B**) Memory performance in the A–B′ and A–A′ test formats of the forced-choice MST. Error bars reflect standard errors. * indicates *p* < 0.05.

Performance on the forced-choice MST was significantly better for the A–A′ test format compared to the A–B′ test format, *t*(42) = 5.660, *p* < 0.001 (see Fig. 1B). This finding supported the hypothesis that participants would have greater accuracy identifying targets paired with corresponding lures than targets paired with non-corresponding lures. Performance was above chance for both the A–A′ test format, *t*(42) = 25.992, *p* < 0.001, and A–B′ test format *t*(42) = 17.810, *p* < 0.001.

Participants made faster recognition memory judgments on A–A′ test trials (*M* = 2119.91, *SE* = 109.67) than A–B′ test trials (*M* = 2382.21, *SE* = 131.24), *t*(42) = -3.883, *p* < 0.001. To assess the effect of accuracy on reactions times, we conducted a 2 Test Format (A–A′, A–B′) × 2 Accuracy (Correct, Incorrect) repeated-measures ANOVA. Reaction times were faster for the A–A′ test format (*M* = 2334.72, *SE* = 131.46) than the A–B′ test format (*M* = 2503.95, *SE* = 149.19), *F*(1, 42) = 4.576, *p* = 0.038. Additionally, reaction times were faster for correct responses (*M* = 2160.56, *SE* = 106.93) than incorrect responses (*M* = 2678.11, *SE* = 167.25), *F*(1, 42) = 44.649, *p* < 0.001. Test format did not significantly interact with response accuracy to impact reaction times, *F*(1, 42) = 2.922, *p* = 0.095.

### A–B′ test format ERP results

**Figure 2:**
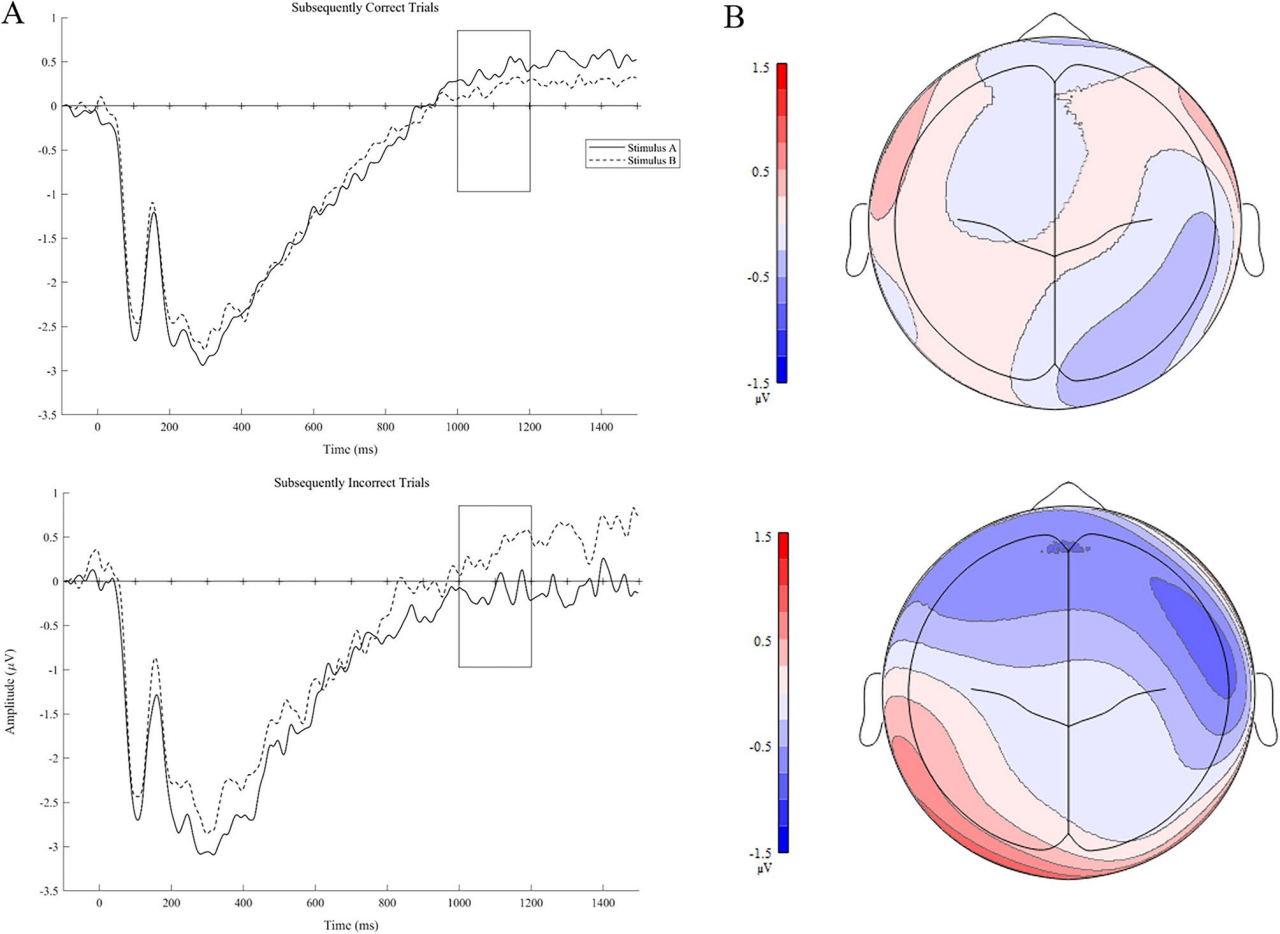
(**A**) Grand mean waveforms for subsequently correct and incorrect A–B′ trials elicited to the A stimulus and original version of the B stimulus presented at encoding. Grand mean waveforms were averaged across all analyzed frontal electrodes because the Accuracy × Stimulus interaction was significant for the frontal plane. These waveforms are not baseline corrected; however, all analyses were conducted on baseline-corrected mean amplitudes. (**B**) Topographical plots illustrating the difference between neural activity elicited to the A stimulus and the original version of the B stimulus at encoding for subsequently correct and incorrect A–B′ trials.

For the A–B′ test format, the omnibus ANOVA revealed a significant Time × Accuracy × Stimulus × Coronal Plane interaction, *F*(12, 348) = 3.12, *p* = 0.016. Neither the main effect of Accuracy nor other interactions with Accuracy were significant, *ps* ≥ 0.104. To assess the interaction, follow-up analyses were conducted separately for each time window. No main effect of or interaction with accuracy were observed in the 400–600 or 600–800 ms time windows, *ps* ≥ 0.266. A main effect of accuracy, which indicates a subsequent memory effect, was observed in the 800–1000 ms window, *F*(1, 29) = 4.227, *p* = 0.049. The amplitude elicited to subsequently correctly recognized stimuli (*M* = 0.471, *SE* = 0.077) was more positive relative to subsequently incorrectly recognized stimuli (*M* = 0.341, *SE* = 0.079). All interactions with accuracy were not significant, *ps* ≥ 0.284. For the 1000–1200 ms window, there was a significant  Accuracy × Stimulus × Coronal Plane interaction, *F*(4, 116) = 6.024, *p* = 0.003. All other main effects and interactions were not significant, *ps* ≥ 0.176. Follow-up 2 Accuracy × 2 Stimulus analyses were conducted along the coronal plane averaging across the sagittal plane. The Accuracy × Stimulus interaction was significant across frontal electrodes, *F*(1, 29) = 7.039, *p* = 0.013 (see Fig. 2). Consistent with the central hypothesis of the current research, follow-up *t*-tests revealed that there was a trend for mean amplitudes to be higher to the A stimulus (*M* = 0.440, *SE* = 0.134) than the B stimulus (*M* = 0.220, *SE* = 0.161) when individuals subsequently correctly identified the target (A), although this finding was not reach the conventional threshold of statistical significance, *t*(29) = 2.033, *p* = 0.051. In contrast, mean amplitudes were significantly higher to the B stimulus (*M* = 0.261, *SE* = 0.205) than the A stimulus (*M* = − 0.150, *SE* = 0.186) when individuals subsequently responded incorrectly by selecting the non-corresponding lure (B), *t*(29) = − 2.248, *p* = 0.032. The Accuracy × Stimulus interaction remained significant across fronto-central electrodes, *F*(1, 29) = 5.330, *p* = 0.028. Similar to the pattern across the frontal electrodes, mean amplitudes were higher to the B stimulus (*M* = 0.546, *SE* = 0.187) than the A stimulus (*M* = 0.062, *SE* = 0.201) when individuals subsequently responded incorrectly by selecting the non-corresponding lure (B), *t*(29) = − 2.507, *p* = 0.018. However, the difference between stimuli was not significant for subsequently correct trials, *t*(29) = 0.663, *p* = 0.512. Neither the main effect of Accuracy nor the Accuracy × Stimulus interaction were significant across the central, centro-parietal, or parietal leads, *ps* ≥ 0.058.

### A–A′ test format ERP results

**Figure 3:**
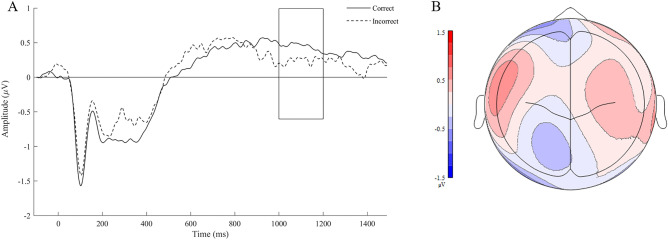
(**A**) Grand mean waveform for subsequently correct and incorrect A–A′ trials. Grand mean waveforms were averaged across all analyzed electrodes due to observing a main effect of Accuracy. These waveforms are not baseline corrected; however, all analyses were conducted on baseline-corrected mean amplitudes. (**B**) Topographical plot illustrating the difference between subsequently correct and incorrect A–A′ trials.

For the A–A′ test format, the omnibus ANOVA revealed a significant Time × Accuracy interaction, *F*(3, 69) = 5.217, *p* = 0.005. Neither the main effect of nor interactions with Accuracy were significant, *ps* ≥ 0.13. To assess the interaction, follow-up analyses were conducted separately for each time window. No main effect of or interaction with accuracy were observed in the 400–600, 600–800, or 800–1000 ms time windows, *ps* ≥ 0.148. The Time × Accuracy interaction likely emerged due to a trend for a main effect of accuracy in the 1000–1200 ms window, *F*(1, 23) = 3.573, *p* = 0.071 (see Fig. 3). Consistent with previous research on the subsequent memory effect, the amplitude elicited to subsequently correctly recognized stimuli (*M* = 0.404, *SE* = 0.078) tended to be more positive relative to subsequently incorrectly recognized stimuli (*M* = 0.200, *SE* = 0.116). No interactions with Accuracy were significant, *ps* ≥ 0.410.

## Discussion

The present study evaluated whether encoding variability, as indexed by ERPs, accounted for behavioral performance on the forced-choice MST. Consistent with previous research^7–9^, behavioral results from the forced-choice MST showed that performance was higher on the the A–B′ test format than the A–A′ test format. In previous research, the MINERVA2 computational model^7^ suggested that encoding variability may account for low performance on the A–B′ test format. Specifically, if the B stimulus was encoded more effectively than the A stimulus, the presentation of the B′ stimulus at retrieval may elicit a stronger global match than the A stimulus leading to its selection. A recent eye tracking study provided support for this notion; errors on the A–B′ test format were associated with more fixations to the B stimulus than the A stimulus at encoding^9,10^.

The primary goal of the current study was to determine whether ERPs recorded at encoding would account for errors on the forced-choice MST. Because SMEs are characterized by subsequently recognized stimuli eliciting a more positive amplitude response at encoding relative to subsequently forgotten stimuli^16,17^, we hypothesized that incorrect A–B′ trials would be associated with a higher amplitude response at encoding to the B stimulus than the A stimulus. In contrast, correct A–B′ trials would be associated with a higher amplitude response at encoding to the A stimulus than the B stimulus. Our data were consistent with this hypothesis over frontal and fronto-central leads 1000–1200 ms poststimulus onset. This finding further supports the role of encoding variability in forced-choice mnemonic discrimination, especially when a target is paired with a non-corresponding lure. Additionally, this finding contributes to current understanding of the late-onset frontal SME, which has been associated with elaborative and associative encoding as well as the subsequent memory for context and recollection-based processing (e.g.,^17–21^). Because participants in the current study incidentally encoded items by making indoor/outdoor judgments, these results provide further support for the late frontal SME not being limited to tasks that encourage associative encoding.

The subsequent memory effect for the A–A′ test format was not significant; however, there was a trend toward the amplitude of the response to the A stimulus at encoding being larger when the target was subsequently correctly selected than when the lure was selected 1000–1200 ms poststimulus onset. The lack of a significant result for the A–A′ test format is consistent with research showing that encoding variability exerts less of an influence on the A–A′ test format than the A–B′ test format because A–A′ test trials elicit the retrieval of correlated memory signals^7,22,23^. According to the MINERVA2 simulations conducted by Huffman and Stark^7^, removing encoding variability only slightly improves performance on the A–A′ test format. Eye-tracking findings with the A–A′ test format were only statistically reliable when trials on which participants guessed were excluded from analyses^9,10^.

In conclusion, the current study suggests that encoding variability reliably accounts for errors on the A–B′ test format of the forced-choice MST 1000–1200 ms poststimulus onset. This finding, taken together with previous research, emphasizes the importance of taking encoding variability into account when assessing the processes that underlie mnemonic discrimination. Future research could examine other sources of variability that contribute to mnemonic discrimination, including list length, the similarity between items in the study list, and the number of stimulus features encoded^7^.

## Methods

### Participants

A total of 43 young adults (*M* = 20.35 years; 29 females, 14 males) provided complete behavioral and electrophysiological data. An additional five individuals participated but were excluded due to the E-Prime program crashing during the testing session (*n* = 2), the recording electrodes not making sufficient scalp contact (*n* = 2) and inattention during the task (*n* = 1). Participants were recruited from a database maintained by the Psychology Department and received course credit for participation.

### Forced-choice MST

Participants completed two study-test blocks of the forced-choice MST (see Fig. 1A). Stimuli included a total of 288 pairs of colored images from the Stark Laboratory’s database of stimuli for the MST (http://faculty.sites.uci.edu/starklab/mnemonic-similarity-task-mst/). For each study block, participants incidentally encoded 144 stimuli by making an indoor/outdoor judgment. Participants were encouraged to maintain fixation on the objects. Each stimulus was presented for 2000 ms and preceded by a fixation cross that varied in duration from 500 to 1000 ms in discrete 50 ms intervals. During each test block, individuals identified which of two stimuli was previously encountered. Targets were either paired with a corresponding lure (A–A′) or a non-corresponding lure (A–B′). Each pair remained on the screen until participants made their response via a button press. Each test format included 48 trials per test format per block that were evenly distributed across four levels of mnemonic similarity (i.e., L1–L4 as determined by previous research; e.g., Stark et al.^24^). Retrieval difficulty on the A–B′ test format was accounted for by matching stimuli as a function of mnemonic similarity levels established by previous research (e.g., Stark et al.^24^). Trials were presented in a randomized order during both study and test. Stimuli used as targets and lures were counterbalanced across participants. Participants took a 5 min break between the two study-test blocks.

### Electrophysiological recording and analysis

Continuous EEG data were acquired during the encoding phases from 64 scalp electrodes, left and right mastoid electrodes, and four electrooculogram (EOG) electrodes (two vertical and two horizontal) at a sampling rate of 512 Hz using a BioSemi Active Two system. Data were re-referenced offline to an average reference using Brain Electrical Source Analysis (BESA) Research version 7.0 (MEGIS Software GmbH, Gräfelfing, Germany; https://www.besa.de). Ocular artifacts were corrected using the Ille et al.^25^ algorithm. Trials on which individuals blinked at stimulus onset or during which other non-ocular artifact occurred (e.g., movement or system-related artifact) were removed using hand-editing by editors that were blind to condition labels. Data were high- and low-pass filtered at 0.1 and 30 Hz, respectively. ERPs were epoched with a 100 ms baseline and extended until 1500 ms post-stimulus onset. The artifact scanning tool in BESA was also used to reject trials containing artifacts (amplitude > 120 µV, gradients > 75 µV). Similar to previous research (e.g.,^11,26^), a minimum of 10 trials per condition was required for inclusion in the analysis of ERP data. This criterion resulted in the inclusion of 30 individuals for the A–B′ analyses and 24 individuals for the A–A′ analyses, which is comparable to previous studies (e.g.,^14,15^). For the analyses of the A–B′ test format, the mean number of trials (range) included was 62 (39–79) for the A stimulus on subsequently correct trials, 61 (38–77) for the B stimulus on subsequently correct trials, 21 (10–44) for the A stimulus on subsequently incorrect trials, and 21 (10–43) for the B stimulus on subsequently incorrect trials. For the analyses of the A–A′ test format, the mean number of trials included was 66 (50–80) for subsequently correct trials and 17 (10–32) for subsequently incorrect trials. Baseline corrected mean amplitudes were exported for 200 ms time intervals ranging from 400 to 1200 ms, consistent with previous research on the subsequent memory effect (Friedman and Trott 2000). ERPs were analyzed from 35 electrode sites (F5, F3, F1, Fz, F2, F4, F6, FC5, FC3, FC1, FCz, FC2, FC4, FC6, C5, C3, C1, Cz, C2, C4, C6, CP5, CP3, CP1, CPz, CP2, CP4, CP6, P5, P3, P1, Pz, P2, P4, P6). Data from the A–B′ test format were assessed separately for each time window using a Time (400–600 ms, 600–800 ms, 800–1000 ms, 1000–1200 ms) × Subsequent Accuracy (Correct, Incorrect) × 2 Stimulus (A, B) × 5 Coronal Plane (F, FC, C, CP, P) × 7 Sagittal Plane (5, 3, 1, z, 2, 4, 6) repeated-measures ANOVA and appropriate follow-up analyses. The analysis for the A–A′ test format was the same except Stimulus was not included as a factor. Only a main effect of or interactions with accuracy are reported.

### Ethical approval

All procedures were approved by the Institutional Review Board at Christopher Newport University and performed in accordance with the Declaration of Helsinki. A verbal and written description of the study was provided to participants, and participants provided written informed consent.

## Data Availability

The data that support the findings of this study are available from the corresponding author L. R. upon reasonable request.
